# Adolescent firearm injury trends at a Level I trauma center in Washington State, USA, 2011–2021

**DOI:** 10.1186/s12887-025-05816-0

**Published:** 2025-06-05

**Authors:** Erika Marts, Frederick P. Rivara, N. Jeanie Santaularia, Ali Rowhani-Rahbar

**Affiliations:** 1 Department of Epidemiology, School of Public Health, University of Washington, Seattle, USA; 2 Firearm Injury & Policy Research Program, Department of Pediatrics, School of Medicine, University of Washington, Seattle, USA; 3 Department of Pediatrics, School of Medicine, University of Washington, Seattle, USA

**Keywords:** Firearm injury, Adolescents, Washington State, Trends

## Abstract

**Background:**

Firearm-related harm is a pressing public health problem in the United States, particularly among adolescents. While informative, national level data may mask variations in firearm injury incidence across and within states.Publicly available firearm injury data may be limited or unavailable, highlighting the importance of analyzing more granular data to develop or refine tailored interventions shaped by the unique needs and contexts of individual communities.

**Methods:**

This retrospective cohort study used data from a Level I Trauma Center and death records to examine adolescent firearm injury trends in King County, Washington from 2011 to 2021. We calculated incidence rates per 100,000 population for each year of the study period and examined differences by fatality, intent, race, ethnicity, age, and sex.

**Results:**

Annual incidence rate of overall firearm injury significantly increased during the study period from 18.1 to 24.9 per 100,000 persons (*p* = 0.003); we observed the highest rate in 2020 (26.5 per 100,000 persons) and the lowest in 2013 (11.5 per 100,000 persons). Nonfatal firearm injuries comprised 71.4% of our sample and had the highest rate in 2020 (18.7 per 100,000 persons), while the rate of fatal firearm injuries was highest in 2021 (8.0 per 100,000 persons). Assault-related firearm injuries were most frequent (73.7%), were primarily nonfatal (81.7%), and significantly increased during the study period (*p* < 0.001). The rate of self-inflicted injuries surpassed that of unintentional injuries during the study period and the majority were fatal (92.5%). Overall firearm injury incidence rates were highest among adolescents aged 18–19, males, and individuals from racial or ethnic minority groups.

**Conclusion:**

The rate of firearm injuries among King County adolescents significantly increased between 2011 and 2021. Our study identified a disproportionate burden of firearm-related harm among King County adolescents who are Black, American Indian or Alaska Native, Native Hawaiian or Other Pacific Islander, Latine, male, and aged 18–19, highlighting disparities in firearm injury by demographic subgroups. These findings may assist the development of local evidence-informed prevention and intervention strategies and guide the implementation of existing interventions among adolescents disproportionately impacted by firearm-related harm.

## Background

Firearm violence is a critical and growing public health problem in the United States, particularly among adolescents. Nationally, approximately 3,000 adolescents between the ages of 10 and 19 are killed by firearms each year [1],with nonfatal injuries being at least twice as common as fatal injuries [2]. Compared to other penetrating trauma mechanisms (e.g., cutting, piercing injuries), firearm injuries are more likely to result in greater injury severity and healthcare utilization among adolescents for both critical and noncritical injuries [3]. Adolescence represents a crucial developmental period for brain maturation, yet adolescents experience the most exposure to violence than any other age group [4]. Exposure to violence during this formative stage increases the risk of repeated violent victimization later in life and is associated with a wide range of short and long-term adverse health outcomes [5]. Following a firearm injury, adolescent survivors are significantly more likely than their non-injured counterparts to experience severe mental health impacts, such as post-traumatic stress symptoms, depression, anxiety, and suicidal thoughts [6]. Substantial increases in self-reported pain, as well as psychiatric and substance use disorders, have been noted within a year after a firearm injury and are associated with mental health impacts on a survivor’s family [7].

While national-level data are vital for understanding the burden of firearm-related harm (e.g., firearm injuries and deaths) among adolescents across the United States, they may mask variations by region. Firearm injury trends, hospital resource utilization, patient demographics, and intent of injury differ by U.S. region and state [8, 9]. Additionally, rates of firearm injury have also notably varied within states and between rural and urban counties [10, 11]. Publicly available datasets, such as those provided by the Centers for Disease Control and Prevention (e.g., CDC WONDER and CDC WISQARS) have some limitations including data suppression within certain subgroups or by geographic regions (e.g., at the county or city-level), and nonfatal firearm injury data may be limited or unavailable [12, 13]. Examining the distribution and trends of firearm injuries on a more geographically granular scale, such as at the county level, is crucial for obtaining precise estimates of firearm injury rates (especially nonfatal) and thus identifying communities and groups disproportionately impacted by firearm-related harm. These findings can inform the development of effective interventions that are tailored to the local context, are more relevant or acceptable to community members, and ultimately may be more effective in decreasing the burden of firearm-related harm in a region.

We sought to characterize adolescent firearm injury trends in King County, Washington (WA), which is the 12th most populous county in the United States [14]. Specifically, we examined trends in firearm injury incidence rates among adolescents aged 10 to 19; described firearm injury by fatality, intent, age, race, ethnicity, and sex; and explored differences in firearm injury incidence rates by fatality, intent, and within demographic subgroups.

## Methods

### Study design

This retrospective cohort study was conducted to identify and describe trends in firearm injuries among adolescents aged 10 to 19 years residing in King County who were included in the regional trauma center hospital-based trauma registry for a nonfatal firearm injury or who sustained a fatal firearm injury during the study period. The exposure of interest in this study was time; we included injury data from January 1, 2011, through December 31, 2021. The outcome of interest was firearm injury, which was further categorized by fatality and intent. To identify nonfatal firearm injuries among our sample and classify them by intent, we used the *International Classification of Diseases, Ninth Revision, Clinical Modification (ICD-9-CM)* external cause codes for trauma registry records through October 2015 (unintentional: 922.0–922.9, self-harm: 955.0–955.4, assault: 965.0–965.4, legal intervention: 970, undetermined: 985.0–985.4) and *International Classification of Diseases, Tenth Revision, Clinical Modification (ICD-10-CM) *codes for records in subsequent months/years (unintentional: W32-W34, self-harm: X72- X74, assault: X93-X95, legal intervention: Y35.0, undetermined: Y22-Y24), using specific codes within intent categories that indicated a powder firearm injury. The misclassification of intent among nonfatal firearm injuries, particularly the coding of assault-related injuries as accidental/unintentional in hospital discharge data, is greatly reduced using trauma registry data given the high sensitivity associated with coding by trauma registrars [15]. We identified fatal firearm injuries and classified their intent using *International Statistical Classification of Diseases and Related Health Problems, Tenth Revision (ICD-10)* external cause codes as captured from death certificates (unintentional: W32-W34, self-harm: X72- X74, assault: X93-X95, legal intervention: Y35.0, undetermined: Y22-Y24), which are determined by physician Medical Examiners as all violent deaths in the county are reviewed by the King County Medical Examiner’s Office.

This study explored differences in firearm injury trends by fatality, intent, and four demographic variables: age, sex, race, and ethnicity. Age was categorized into three groups, 10–14, 15–17, and 18–19, based on prior evidence that injury intent differs between adolescent age categories [2, 11, 16]. Racial and ethnic minority groups (e.g., Black, Latine, American Indian or Alaska Native, Native Hawaiian or Other Pacific Islander adolescents), as well as males, are disproportionately impacted by firearm-related harm in the United States [17–20], therefore this analysis also examined differences by sex, race, and ethnicity. Adolescents were classified as living in rural or urban areas based on their residence zip code and its assigned Rural–Urban Commuting Area code (RUCA), which designates census tracts as urban or rural based on measures of population density, urbanization, and daily commuting (zip codes with a RUCA code of 4.0, 4.2, 5.0, 5.2, 6.0, 6.1, 7.0, 7.2, 7.3, 7.4, 8.0, 8.2, 8.3, 8.4, 9.0, 9.1, 9.2, 10.0, 10.2, 10.3, 10.4, 10.5, or 10.6 were classified as rural) [21].

This study received approval by the University of Washington Institutional Review Board.

### Setting

King County, Washington has a population of approximately 2.3 million people, of whom about 11% are between 10 to 19 years [14]. The region encompasses both urban and rural areas, with roughly 90% of the population living in cities [22]. Demographically, the population is 54% White, 20% Asian, 11% Hispanic, 7% Black, and 7% multiracial.

### Study subjects

Nonfatal injuries were identified through the trauma registry of Harborview Medical Center (HMC), the only Level I adult and pediatric trauma center in Washington State and the surrounding states (i.e., Alaska, Idaho, Montana, and Wyoming), which included information on anyone who presented to the trauma center with a gunshot wound during the study period. Fatal injuries were captured from death certificates from the Washington State Department of Health Center for Health Statistics. Participants were included if they were between the ages of 10 and 19 years and resided in King County at the time of injury. Participants who were included in the trauma registry but indicated living in a different county or were residents of King County but presented to other medical centers for a nonfatal injury were not included in this sample. A total of 490 adolescents aged 10–19 residing in King County who sustained a fatal or nonfatal firearm injury between 2011 and 2021 were included in the analytical sample.

### Data collection

Data from the trauma registry and death certificates provided the date of injury, victim’s age at the time of injury, county where the injury occurred, state of residence, county of residence (if in Washington), race, ethnicity, and sex. Census data were obtained from the American Community Survey (ACS) 5-year estimates detailed tables to ascertain the population size of adolescents aged 10 to 19 by age, sex, race, and ethnicity in King County during the study period. Participants in the trauma registry with the same medical record number, a documented firearm injury based on *ICD-9-CM*/*ICD-10-CM* codes, but with a different arrival date than the first documented firearm injury between 2011 and 2021 were classified as experiencing a subsequent firearm injury. We excluded fatal injuries from the trauma registry to avoid capturing duplicate fatal firearm injury records from death certificates.

### Statistical analysis

Data were analyzed using R 4.3.2. Descriptive statistics were calculated to characterize the distribution of firearm injuries by fatality, intent, rurality, age, sex, race, and ethnicity. To examine trends in overall firearm injuries from 2011–2021, we calculated incidence rates per 100,000 population for each year of the study period using counts of injury and population data for King County. Incidence rates were calculated using the epiR package. We plotted incidence rates per year and their corresponding 95% confidence intervals to visualize temporal trends in overall firearm injuries. To calculate incidence rate ratios over time and their corresponding 95% confidence intervals, we fitted a Poisson regression model using counts of firearm injury as the outcome, year as a categorical variable (dummy variable) with 2011 as the reference year, and the population of adolescents aged 10–19 in King County between 2011 and 2021 as the offset term. To test the significance of the time trend, we fitted a Poisson regression model using counts of firearm injury as the outcome, year as an ordered categorical variable, and the population of adolescents aged 10–19 in King County between 2011 and 2021 as the offset term and compared this model to a null model using a likelihood ratio test. A *p*-value less than an alpha level of 0.05 was considered statistically significant.

We calculated incidence rates of firearm injury per year by each of our variables of interest (i.e., fatality, intent, age, sex, race, and ethnicity) and plotted them to visualize differences over time within groups. We visualized incidence rates by demographics using three-year rolling averages to stabilize lines for each subgroup, particularly those with small counts. We then fitted a separate Poisson regression model for each variable of interest using counts of firearm injury as the outcome, year as a continuous variable, an interaction term between year and our fatality, intent, and demographic variables, and the population of adolescents aged 10–19 in King County between 2011 and 2021 as the offset term. We tested the statistical significance of each interaction to determine if there were significant differences in trend between subgroups using a likelihood ratio test, comparing the models that included the interaction terms with the models that did not.

## Results

### Characteristics of the sample

There were 490 firearm injuries captured among King County adolescents aged 10 to 19 years in the trauma registry and death certificates between 2011 and 2021. In the total sample, adolescents aged 18–19 constituted the largest proportion of overall firearm injuries during the study period (53.1%), followed by adolescents aged 15–17 (39.0%) (Table 1). Overall firearm injuries were most frequently assault-related (73.7%), followed by self-inflicted (13.7%) and unintentional (8.8%). Among our sample, 84.9% of all firearm injuries were sustained by male adolescents, 48.6% were among Black adolescents, and 27.6% were among Latine adolescents. During the study period, 97.6% of overall firearm injuries were among urban adolescents. Within the study population, 11 individuals sustained a subsequent firearm injury; all but one of these injuries were nonfatal.

**Table 1:** Demographic characteristics of King County adolescents 10–19 with firearm injuries, 2011–2021

	Fatal (*N* = 140)	Nonfatal (*N* = 350)	Overall (*N* = 490)
Age			
10–14	13 (9.3%)	26 (7.4%)	39 (8.0%)
15–17	53 (37.9%)	138 (39.4%)	191 (39.0%)
18–19	74 (52.9%)	186 (53.1%)	260 (53.1%)
Race			
Asian	10 (7.1%)	8 (2.3%)	18 (3.7%)
Black	33 (23.6%)	205 (58.6%)	238 (48.6%)
Multi-racial	11 (7.9%)	10 (2.9%)	21 (4.3%)
American Indian or Alaska Native	4 (2.9%)	3 (0.9%)	7 (1.4%)
Native Hawaiian or Other Pacific Islander	4 (2.9%)	6 (1.7%)	10 (2.0%)
Not documented	1 (0.7%)	22 (6.3%)	23 (4.7%)
White	77 (55.0%)	96 (27.4%)	173 (35.3%)
Ethnicity			
Hispanic or Latine	67 (47.9%)	68 (19.4%)	135 (27.6%)
Not Hispanic or Latine	73 (52.1%)	282 (80.6%)	355 (72.4%)
Sex			
Female	22 (15.7%)	52 (14.9%)	74 (15.1%)
Male	118 (84.3%)	298 (85.1%)	416 (84.9%)
Intent			
Assault	75 (53.6%)	286 (81.7%)	361 (73.7%)
Legal intervention	2 (1.4%)	5 (1.4%)	7 (1.4%)
Self-inflicted	62 (44.3%)	5 (1.4%)	67 (13.7%)
Undetermined	1 (0.7%)	11 (3.1%)	12 (2.4%)
Unintentional	0 (0%)	43 (12.3%)	43 (8.8%)
Rurality			
Rural	12 (8.6%)	0 (0%)	12 (2.4%)
Urban	128 (91.4%)	350 (100%)	478 (97.6%)

### Firearm injury by fatality and intent

In our sample, 350 (71.4%) firearm injuries were nonfatal, and nonfatal firearm injuries were 2.36 times more common than fatal firearm injuries during the study period (95% CI: 1.94, 2.88). Among nonfatal firearm injuries, 53.1% occurred among individuals aged 18–19, 58.6% among Black adolescents, 19.4% among Hispanic or Latine adolescents, 85.1% among males, and all (100%) were among urban adolescents. Among fatal firearm injuries, 55.0% were sustained by White adolescents, 47.9% by Hispanic or Latine adolescents, 84.3% by males, and 8.6% were among rural adolescents.

Among adolescents with assault-related firearm injuries, most were Black adolescents (55.4%), aged 18–19 (54.8%), and males (84.8%). 79.2% of assault-related firearm injuries were nonfatal. Among adolescents with self-inflicted firearm injuries, 67.2% were White, 47.8% were 15 to 17 years old, 46.3% were Latine, 86.6% were male, and 11.9% lived in rural King County. Adolescents aged 15 and older comprised 88.3% of unintentional firearm injuries; additionally, among unintentional firearm injuries, 81.4% involved males, and 51.2% were among Black adolescents.

### Description of trend

**Fig. 1:**
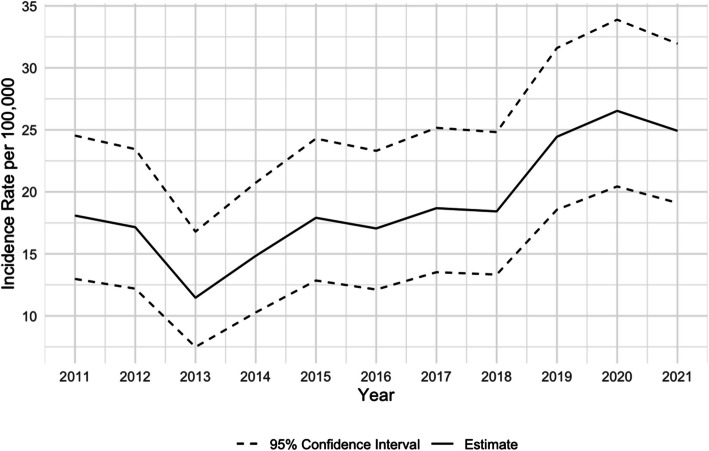
Rates per 100,000 persons of overall firearm injury among King County adolescents 10–19, 2011–2021

**Fig. 2:**
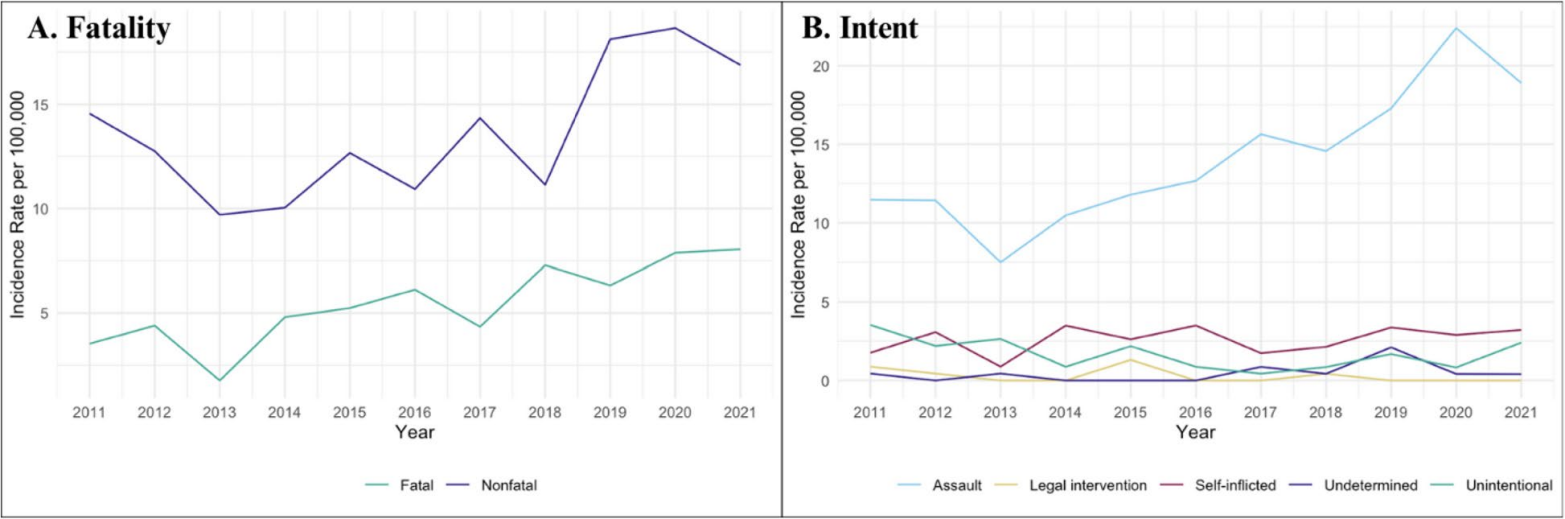
Characteristics of firearm injury: Rates per 100,000 population by fatality and intent, 2011–2021

The annual incidence rate of overall firearm injury significantly increased from 18.1 per 100,000 persons in 2011 to 24.9 per 100,000 persons in 2021 (Fig. 1) (*p* = 0.003). Between 2011 to 2021, the annual rate of overall firearm injury increased by about 6.1% on average (*p* < 0.001). Annual rates of nonfatal firearm injury and fatal firearm injury increased from 2011 to 2021 (14.6 to 16.9 and 3.5 to 8.0 per 100,000 persons, respectively; Fig. 2) (*p* = 0.33). We observed the lowest annual rates of overall, nonfatal, and fatal firearm injuries among our sample in 2013 (11.5, 9.7, 1.8 per 100,000 persons, respectively). The highest annual rate of nonfatal firearm injuries occurred in 2020 (18.7 per 100,000 persons), while the annual fatal firearm injury rate was highest in 2021 (8.0 per 100,000 persons). The highest overall firearm injury rate was observed in 2020 (26.5 per 100,000 persons).

**Fig. 3:**
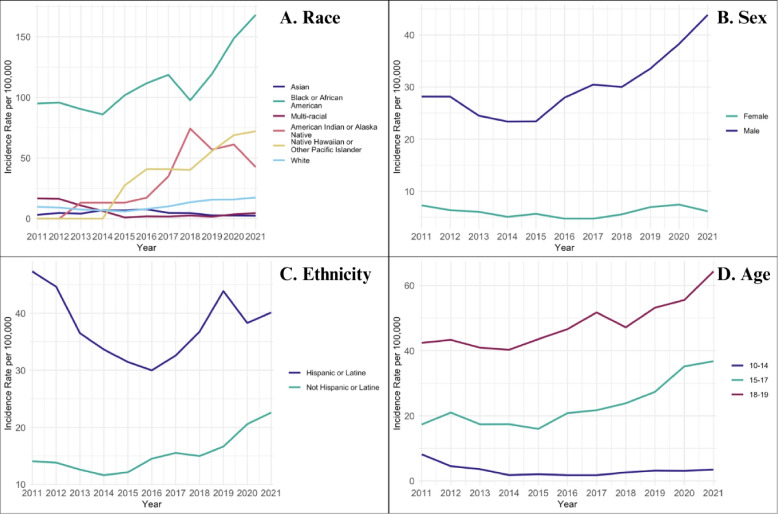
Sociodemographic characteristics of adolescents with firearm injury: Rates* per 100,000 persons by race, sex, ethnicity and age, 2011–2021. *Rates presented as three-year rolling averages

In our sample, differences in trend between subgroups were statistically significant by intent (*p* = 0.01) and race (*p* = 0.01). The annual rate of assault-related firearm injuries increased by approximately 8.7% on average (*p* < 0.001), while the annual rate of unintentional injuries decreased by an average of 8.0% each year between 2011 and 2021 (*p* = 0.001). While overall firearm injury incidence rates were highest among Black adolescents, we observed the greatest average increase in the rate of overall firearm injury rates per year on the relative scale among adolescents who were Native Hawaiian or Pacific Islander (IRR = 1.24, 95% CI: 1.02, 1.71), American Indian or Alaska Native (IRR = 1.15, 95% CI: 0.83, 1.58), and White (IRR = 1.11, 95% CI: 1.02, 1.38) (Fig. 3). Overall firearm injury rates were highest among adolescents aged 18 to 19, males, and Latine adolescents throughout the study period; however, these differences in trend between age (*p* = 0.22), sex (*p* = 0.20), and ethnicity (*p* = 0.19) subgroups were not statistically significant.

## Discussion

Our study identified a significant increase in the annual rate of overall firearm injuries among King County adolescents between 2011 and 2021. This finding is consistent with previous research indicating that despite overall pediatric emergency department visits declining among this age group, firearm injuries have been increasing in the past decade [17].

In our sample, assault accounted for most firearm-related injuries sustained during the study period, and more than 75% of assault-related injuries in our sample were nonfatal. Assault-related firearm injuries were most common among adolescents who were older (aged 18–19), Black, or male. These findings align with similar research conducted nationally and within other trauma centers regarding the distribution of intent and fatality of firearm injuries among this age group and by demographic group [2, 17]. Assault-related firearm injuries have been strongly associated with high levels of social vulnerability, a measure that incorporates the compounding effects of socioeconomic status, household composition and disability, minoritized status and language, housing type, and transportation [23]. In King County, prior work has found that rates of firearm injury were higher in areas with greater social vulnerability [24]. Since the end of the study period in 2021, Harborview Medical Center has launched a hospital-based violence intervention program, which connects victims of gun violence, their families, and their communities with individualized community services such as counseling, temporary emergency housing or relocation, and training to promote employment and educational goals [25, 26]. Similar programs have demonstrated reductions in repeat violent injury, criminal legal system involvement, and perpetration of violent behaviors [27].

We observed a concerning increase in the rate of self-inflicted firearm injuries among adolescents during the study period, of which 92.5% were fatal, emphasizing the lethality of firearms as a means for suicide [28]. Washington State passed a secure storage law on July 1, 2019 [29]; such laws (specifically, child access prevention [CAP] laws) have been associated with reductions in suicide and self-inflicted firearm injuries, unintentional shootings, and firearm homicides and assaults among children and adolescents [30]. Future work could examine how policies, programs, and practices have impacted the rate of firearm injuries among adolescents in King County and Washington State. Access to firearms in the home increases suicide risk among adolescents, highlighting the importance of secure storage (locked, unloaded, and separate from ammunition) in homes where adolescents are present [31, 32]. Firearm lock distribution programs, which provide safety education and free locking devices to gun owners in a community, have shown promise in improving storage practices [33]. Individuals experiencing indicators of economic instability (e.g., receiving food stamps, precarious employment, etc.) have been found to be more likely to store firearms loaded and unlocked, suggesting secure storage efforts should prioritize low-resource communities [34]. After 2021, Public Health-Seattle & King County’s Lock-It-Up program was expanded to improve lock distribution, public messaging, and support for clinical and community organizations [35]. Evaluations of this effort and similar programs should explore the distribution of locking devices across various socioeconomic, geographic, and demographic groups to ensure equitable impact. Similarly, several community-based organizations in King County have actively engaged in implementing different forms of community violence intervention programs in recent years. The King County Regional Office of Gun Violence Prevention and Regional Peacekeepers Collective have provided resources to support those efforts [36]. Measuring the collective impact of these investments on the health, safety, and well-being of adolescents and communities in different parts of King County will be an important next step.

Our findings also reflected those of previous work that highlighted the disproportionate burden of firearm-related harm, particularly due to assault, among older adolescents, adolescents in racial and ethnic minoritized groups, and males [1, 2, 17–20]. Notably, our study identified a significant relative annual average increase in the rate of firearm injury among Native Hawaiian or other Pacific Islander and White adolescents. We additionally observed an increase in the rate of overall firearm injuries among American Indian or Alaska Native and Black adolescents, although this increase was not statistically significant. Due to the small numbers within some racial subgroups, such as Native Hawaiian or other Pacific Islander and American Indian or Alaska Native, this study has limited statistical power to detect significant differences in trend between subgroups. We presented incidence rates for all demographic subgroups using rolling averages to ensure their inclusion, acknowledging the importance of representing all adolescents in the sample and highlighting racial disparities in firearm-related harm, especially given the historical exclusion of some groups from observational research due to small sample sizes [37, 38]. We also identified higher overall firearm injury rates among Latine adolescents compared to non-Latine adolescents, although the difference in trend between ethnic groups was not significant. Several individual, community, and structural-level risk factors have been identified in the literature to explain disparities in the distribution of firearm-related harm, including economic disadvantage and structural racism [39].

To our knowledge, this study is the first to examine trends in firearm injuries among adolescents aged 10–19 in King County and identify how these trends differ by fatality, intent, and demographic factors. These data may aid community members and decision makers in designing, implementing, and refining tailored interventions that are culturally relevant, context-specific, and widely accepted by the community. Such interventions may more effectively alleviate the harmful impacts of firearm violence on victims—both those who have lost their lives and survivors—as well as their families and communities across the region. Preventing firearm violence and supporting adolescent survivors of firearm injury is critical to stop the cycle of community firearm violence, support the healthy development of adolescents, and mitigate the wide range of adverse mental health impacts experienced by survivors and adolescents who were exposed to violence throughout the life course [4, 40, 41].

This study has some limitations. While misclassification of intent for nonfatal firearm injuries is less common in trauma registry data, coding of intent in death certificates may also have some inaccuracies, particularly by undercounting unintentional deaths or those due to legal intervention [42, 43]. However, in King County the physician Medical Examiner sees all patients who die from firearms and is responsible for the classification of intent after full investigation, lessening the risk of misclassification. While *ICD-10-CM* codes allow for a clearer identification of nonfatal injuries due to nonpowder firearms (e.g., air guns, pellet guns), *ICD-10 *codes may conflate fatal injuries from nonpowder firearms with those from powder firearms [44]. However, fatal injuries from nonpowder firearms are rare, reducing the likelihood of misclassifying nonpowder and powder firearm injuries in fatality data [45]. Our study did not capture adolescents who presented to other hospitals which may introduce ascertainment bias, though as the only Level I trauma center in the region, the hospital treats most firearm injuries in the county. Previous research has found inaccuracies in race and ethnicity coding, particularly for American Indian/Alaska Native, Native Hawaiian/Other Pacific Islander, and Asian individuals, which may affect our results [46–48]. Lastly, while LGBTQ + communities are disproportionately impacted by firearm violence [49], this study used data that are not inclusive of diverse gender identities, limiting our understanding of how gender non-conforming or transgender individuals in King County may be impacted by firearm injury and gun violence.

## Conclusions

The annual rate of firearm injuries among adolescents in King County increased significantly from 2011 to 2021, with disproportionate impacts among males, older adolescents, and adolescents in racial and ethnic minority groups. Increases in overall firearm injury rates were primarily driven by assault-related injuries, most of which were nonfatal and disproportionately impacted Black adolescents. Due to the long-term, harmful impacts associated with firearm injury among survivors [6], access to and connection with mental health care following a firearm injury is critical. This local-level data may aid community-based organizations and other agencies in advocating for additional resources to reduce disparities in firearm-related harm or to implement targeted interventions across new demographic groups or settings. Subsequent work among adolescents in this and other settings could utilize a qualitative or mixed methods approach to incorporate the lived experience of survivors of gun violence and firearm-related harm. Additionally, longitudinal studies following the trajectory of survivors could identify risk factors for repeat firearm injury and improve interventions for reducing the toll of firearm-related harm among adolescents.

## Data Availability

American Community Survey 5-year Data used in the analysis for this study are available from the United States Census Bureau at the following URL: https://www.census.gov/data/developers/data-sets/acs-5year.html. The trauma registry data that support the findings of this study are maintained by Harborview Medical Center, but restrictions apply to the availability of these data, which were used under license for the current study, and so are not publicly available. The death certificate data that support the findings of this study are maintained by the Washington State Department of Health Center for Health Statistics, but restrictions apply to the availability of these data, which were used under license for the current study, and so are not publicly available.

## Abbreviations

WA: Washington
ICD-9-CM: International Classification of Diseases, Ninth Revision, Clinical Modification
ICD-10-CM: International Classification of Diseases, Tenth Revision, Clinical Modification
RUCA: Rural–Urban Commuting Area
HMC: Harborview Medical Center
ACS: American Community Survey
CAP: Child Access Prevention laws
CDC WONDER: Centers for Disease Control and Prevention Wide-ranging ONline Data for Epidemiologic Research
CDC WISQARS: Centers for Disease Control and Prevention Web-based Injury Statistics Query and Reporting System
LGBTQ +: Lesbian, Gay, Bisexual, Transgender, and Queer
